# Breast pseudoaneurysm following tomosynthesis-guided vacuum-assisted biopsy successfully treated with ultrasound-guided thrombin injection: A case report of a rare breast emergency

**DOI:** 10.1016/j.radcr.2026.05.070

**Published:** 2026-06-13

**Authors:** Antonia Berz, Jana Di Vincenzo, Georgia Tsoumakidou, Guillaume Fahrni, Rami Hajri

**Affiliations:** Department of Diagnostic and Interventional Radiology, Lausanne University Hospital and University of Lausanne, Lausanne, Switzerland.

**Keywords:** Breast pseudoaneurysm, Vacuum-assisted breast biopsy, VABB, Ultrasound-guided thrombin injection, Iatrogenic vascular complication

## Abstract

Digital tomosynthesis-guided vacuum-assisted breast biopsy is a standard minimally invasive technique for diagnosing focal breast lesions, with vascular complications such as pseudoaneurysm formation being exceedingly rare. We report the case of a 64-year-old postmenopausal woman who developed a 2.6-cm intramammary pseudoaneurysm in the right breast following digital tomosynthesis-guided vacuum-assisted breast biopsy for high-grade ductal carcinoma in situ adjacent to an invasive carcinoma with choriocarcinomatous differentiation, successfully treated with ultrasound-guided percutaneous thrombin injection, without complications. This case emphasizes the importance of early recognition of post-biopsy pseudoaneurysms to avoid potential hemorrhagic complications. Furthermore, this case demonstrates that ultrasound-guided breast thrombin injection is a safe and effective minimally invasive treatment option and highlights the importance of individualized therapeutic approaches to optimize outcomes.

## Introduction

Digital tomosynthesis-guided and stereotactic vacuum-assisted breast biopsy (VABB) is a well-established, minimally invasive technique for diagnosing focal breast lesions, notably microcalcifications and are generally considered safe, with a very low complication rate, including soft-tissue complications, hematomas and, uncommonly, pseudoaneurysms (PSA) [1].

A PSA, or false aneurysm, results from a focal arterial wall injury, leading to a localized collection of blood that remains in communication with the parent artery through a narrow neck, forming a hematoma with turbulent flow [2,3]. Unlike a true aneurysm, the vessel wall itself is not involved; instead, the PSA is contained by a fibrous capsule composed of fibrin and platelet aggregates, rendering it structurally weaker and making prompt recognition essential to prevent rupture or bleeding [2,3].

To date, to the best of our knowledge, only about 36 cases of breast PSA after biopsy have been documented in the literature, most of them following core-needle biopsy with the majority managed surgically. Here, we report a case of an iatrogenic breast PSA occurring after tomosynthesis-guided VABB in association with a highly vascular tumor subtype (choriocarcinomatous differentiation), successfully treated with ultrasound-guided thrombin injection. This case highlights a rare complication of vacuum-assisted breast biopsy and supports percutaneous thrombin injection as a minimally invasive alternative to the predominantly surgical management described to date.

## Case report

A 64-year-old postmenopausal woman under hormonal substitution with a history of surgically treated vulvar intraepithelial neoplasia 3 (VIN 3) and a positive family history of gynecological cancers was referred to our institution for further evaluation following a positive breast cancer screening. Bilateral screening mammography revealed a 1-cm spiculated mass in the upper outer quadrant of the right breast, in proximity to pleomorphic segmentary micro- and macrocalcifications (Fig. 1A,C), classified as BI-RADS 5 (highly suggestive of malignancy, >95%). In the contralateral breast, an irregular 1-cm mass with fine clustered microcalcifications was detected in the lower inner quadrant (Fig. 1B,D), classified as BI-RADS 4C (high suspicion for malignancy, 50%-94%).

**Fig. 1:**
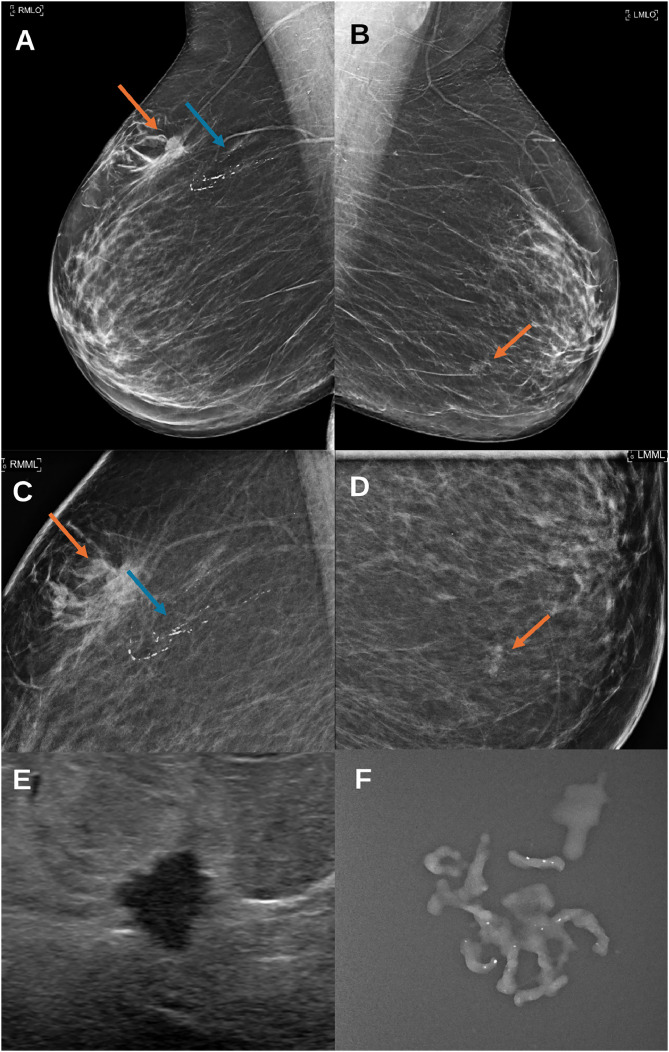
Breast imaging screening demonstrating bilateral breast lesions. Screening mammography (*mediolateral oblique view, first row*) and diagnostic tomosynthesis (*mediolateral view, second row*) revealed in the upper outer quadrant of the right breast (*A, C*) a 1-cm spiculated mass (*orange arrows*) with associated pleomorphic segmental micro- and macrocalcifications (*blue arrows*), classified as BI-RADS 5. In the lower inner quadrant of the left breast (*B, D*), an irregular 1-cm mass with fine clustered microcalcifications (*orange arrow*) was identified, classified as BI-RADS 4C. Targeted US (*E*) of the right breast confirmed a heterogeneous hypoechoic lesion in the upper outer quadrant measuring 1 × 0.7 × 0.7 cm. Specimen radiograph (*F*) of the adjacent pleomorphic segmental micro- and macrocalcifications (*not visible on US*) confirmed adequate sampling of the calcifications.

Targeted US of the right breast confirmed a heterogeneous hypoechoic lesion in the upper outer quadrant, with an echogenic rim and internal color Doppler vascularity, measuring approximately 1 × 0.7 × 0.7 cm and located 4 cm from the nipple (Fig. 1E). An US-guided core biopsy of the lesion using an 18-gauge Biopince BioPince^TM^ automated core biopsy instrument (Argon Medical Devices, INC) was performed in the same session without immediate complications. Histopathological analysis confirmed an invasive carcinoma of no special type (NST), grade 3, triple negative (ER-, PR-, HER2-), with a high proliferative index (Ki-67 80%) and choriocarcinomatous differentiation. To evaluate the adjacent pleomorphic segmentary micro- and macrocalcifications, which were not visible on US, a tomosynthesis-guided VABB was subsequently performed: We used a Hologic Affirm table with the patient in prone position and standard mediolateral compression of the right breast. An initially obtained localizing tomosynthesis acquisition of the right breast allowed target selection of the most suspicious area of the microcalcifications. Local anesthesia of the skin and biopsy trajectory was administered. A 9-gauge Eviva needle (Guided Breast Biopsy Device, Hologic, Inc.) was inserted approximately 7 cm deep using a cranial approach. The needle aperture of 20 mm was positioned exactly in front of the target. A vacuum assisted sample aspiration, yielding 12 tissue samples, without immediate adverse events was performed. Post-biopsy tomosynthesis confirmed adequate retrieval of the calcifications (Fig. 1F). Subsequently a biopsy clip was inserted. Standard post-biopsy manual breast compression was applied for approximately 10 minutes with the patient in the supine position following a compressive dressing with an elastic bandage. The patient underwent clinical evaluation 1 hour after the procedure and was discharged home after an unremarkable examination.

Three days later, a staging breast magnetic resonance imaging (MRI) showed the well-known spiculated lesion in the upper outer quadrant of the right breast corresponding to the biopsy-proven invasive carcinoma (Fig. 4A). The BI-RADS 4C lesion of the lower inner quadrant of the left breast presented a non-mass enhancement, defined as enhancement without a corresponding discrete mass or space-occupying lesion (Fig. 4B). Incidentally, at the site of the VABB in the upper quadrants of the right breast, a large multiloculated intramammary hematoma of approximately 10×7×7.5 cm was observed, responsible for breast swelling and associated with hematoma-related skin edema and infiltration (Fig. 2A, B). Within the hematoma, a well-circumscribed oval lesion measuring 1.4 cm x 1.1 cm in diameter in the axial plane, of mixed signal on T1 and T2, showing avid and early enhancement was identified in the upper inner quadrant, 8.5 cm from the nipple, consistent with the circulating lumen of a PSA, supplied by a feeding artery arising from the internal mammary artery. Retrospectively, the patient reported progressive right breast swelling and discomfort since the biopsy procedure.

**Fig. 2:**
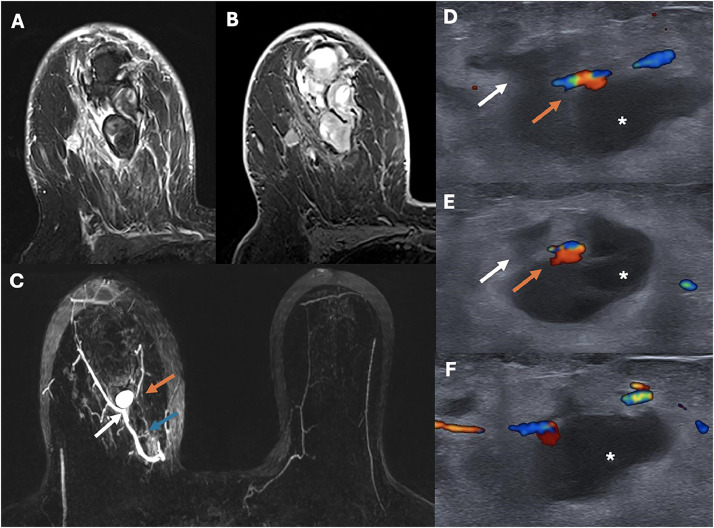
Imaging diagnosis of a breast pseudoaneurysm following vacuum-assisted biopsy. Breast MRI performed 3 days post-VABB showed, on T2-fat sat *(A)* and T1 fat sat *(B)* weighted images, a large multiloculated intramammary hematoma at the biopsy site in the upper quadrants of the right breast, measuring approximately 10×7×7.5 cm. Dynamic contrast enhanced T1-weighted perfusion images (DCE) with subtraction *(C)* revealed a well-circumscribed 1.4 cm oval enhancing lesion (*orange arrow*) consistent with the circulant lumen of a PSA in the right breast, with a narrow neck (*white arrow*) supplied by a feeding artery arising from the internal mammary artery (*blue arrow*). Targeted color Doppler US (*D-F*) showed a 2.6 cm PSA sac with a narrow neck (*white arrow*), a 0.7 cm patent lumen (*orange arrow*) and a thrombosed portion (*).

Given these findings, the patient was referred to our interventional radiology department for targeted color Doppler US evaluation 5 days later. This confirmed a PSA in the right breast, with a patent circulating lumen of 0.7 cm showing marked turbulent flow and a partially thrombosed sac measuring 2.6 cm (Fig. 2C-F). A classic “yin-yang” sign and/or “to-and-fro” waveform were not explicitly documented. The discrepancy in measurements between MRI and ultrasound likely reflects the dynamic character of these lesions, with the MRI demonstrating a larger circulating lumen and the ultrasound performed 5 days later showing reduction of the residual lumen, consistent with progressive thrombosis. Furthermore, differences in patient positioning between MRI and ultrasound examinations may also have contributed, to a lesser extent, to the variation in pseudoaneurysm measurements. A decision was made to treat the PSA with percutaneous thrombin injection. Under strict aseptic conditions, sterile draping and local anesthesia (Lidocaine 1%, 10ml) a 19-gauge spinal needle was advanced under US-guidance into the PSA lumen, followed by slow injection of 2 ml of fibrin sealant (Tisseel^Ⓡ^, Baxter AG). Immediate hyperechoic transformation of the lumen and cessation of blood flow on color Doppler US were observed, indicating successful thrombosis. The needle was then removed, and the patient was discharged home after 1 hour. Follow up color Doppler US at 3 days demonstrated persistent complete thrombosis of the PSA with no residual flow (Fig. 3A, B).

**Fig. 3:**
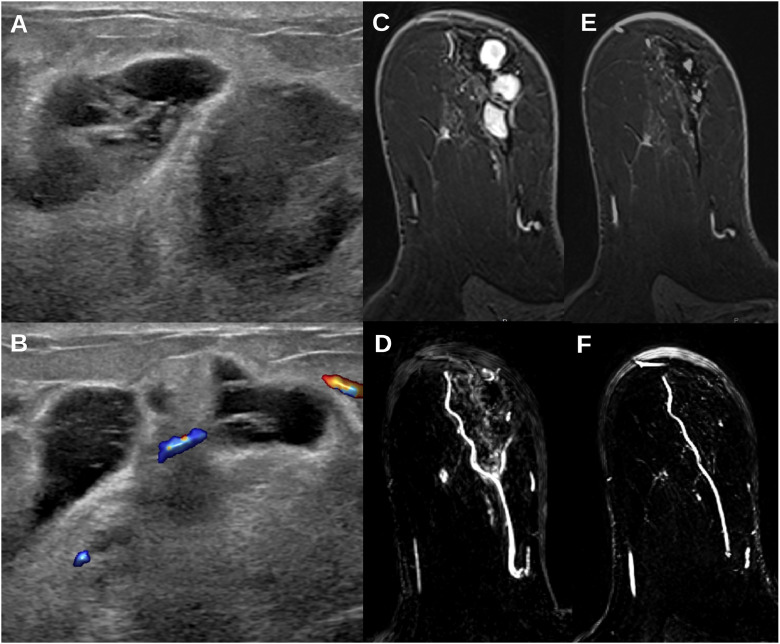
Complete thrombosis of the breast pseudoaneurysm and progressive hematoma resolution after percutaneous thrombin injection*.* Immediately post-treatment B-mode (*A*) and color Doppler US (*B*) demonstrated a large organizing hematoma in the right breast with no residual flow within the lesion, consistent with complete thrombosis following percutaneous thrombin injection. Follow-up MRI with T1-fat sat (*upper row*) and dynamic contrast enhanced T1- weighted perfusion images (DCE) with subtraction (*lower row*) at the 2 months (*C, D*) and 5 months follow-up (*E, F*), showed progressive reduction in the size of the hematoma and no recurrence of the vascular lesion.

Once the PSA had been effectively managed, attention was subsequently directed to the evaluation of the contralateral breast lesion which had no sonographic correlate. Histopathological analysis, performed in tissue samples collected in a second session using the same VABB technique as previously described, without immediate complications, confirmed DCIS of intermediate to high nuclear grade, with cribriform and focally solid architecture, necrosis, calcifications, and with hormone receptor expression (ER 100%, PR 50%).

Follow-up routine MRI, performed 2 and 5 months after initiation of neoadjuvant chemotherapy, demonstrated progressive reduction of the hematoma and no recurrence of the PSA (Fig. 3C-F), and a sustained excellent oncologic response of both breast tumors (Fig. 4C-F). Whole-body FDG PET-CT and brain MRI excluded distant metastases. Genetic testing revealed a BRCA1 (BReast CAncer gene 1) mutation, and the patient is currently scheduled for bilateral mastectomy with right-sided sentinel lymph node resection and simultaneous bilateral adnexectomy.

**Fig. 4:**
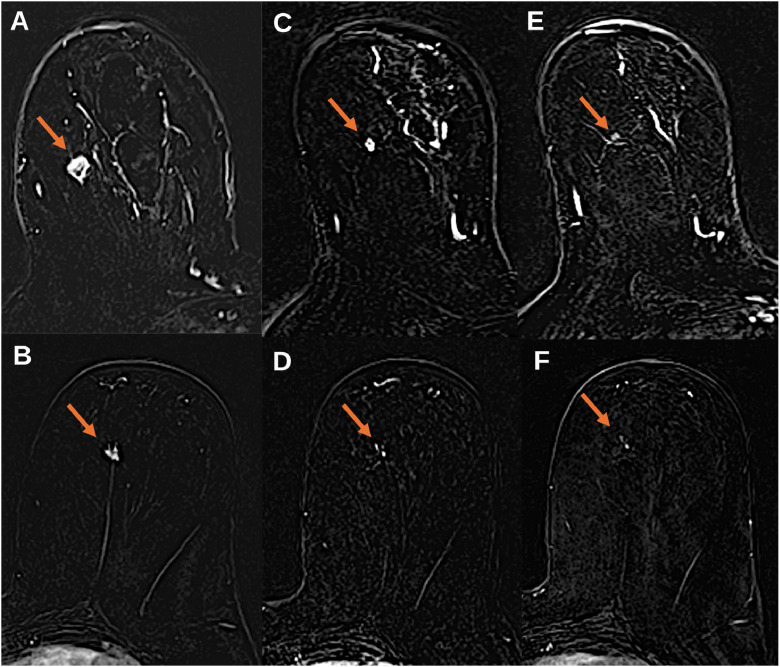
Progressive treatment response of the bilateral breast cancers during neoadjuvant systemic therapy. Dynamic contrast enhanced T1- weighted perfusion images (DCE) with subtraction for the right breast (*upper row*) and the left breast (*lower row*). Baseline MRI (*A, B*) demonstrates the invasive carcinoma in the upper outer quadrant of the right breast (*A*) and the DCIS in the lower inner quadrant of the left breast (*B*). An intermediate MRI performed 2 months after initiation of neoadjuvant systemic therapy (*C, D*) shows partial treatment response of both lesions. A subsequent MRI performed 3 months later, at completion of neoadjuvant therapy (*E, F*), demonstrates an excellent oncologic response in both breasts, classified as BI-RADS 6 (biopsy-proven malignancy).

## Discussion

*Incidence and etiology*Although iatrogenic PSAs following percutaneous punctures are overall uncommon, with an incidence of around 3%, they may occur following arterial punctures involving the cardiac, femoral, visceral, or aortic arteries [3,4]. Breast PSAs are considerably rarer, usually occurring following percutaneous biopsies, with only about 36 cases reported in literature [3,[5], [6], [7], [8], [9], [10], [11], [12], [13], [14], [15], [16], [17], [18], [19], [20], [21], [22], [23], [24], [25], [26], [27], [28], [29], [30], [31], [32], [33], [34], [35], [36], [37], [38], [39]].

This iatrogenic complication can occur regardless of the operator’s experience or technical proficiency [15]. Reported predisposing factors include both procedural aspects (use of large-gauge needles, repeated punctures), and patient-related conditions (coagulopathy disorders, anticoagulant or antiplatelet therapy, advanced age, atherosclerosis, large breast volume, lactation) [1,3,10,38]. Furthermore, the tumor histopathology itself, particularly in highly vascularized lesions, may represent an additional risk factor [32,39].

In our patient, the PSA developed after a 9-gauge VABB of a high-grade ductal carcinoma in situ in proximity to an invasive carcinoma with choriocarcinomatous differentiation, a subtype known for its marked vascularity, in a woman with large breast volume and no other identifiable patient-related risk factors.

### Clinical presentation

A breast PSA may occur immediately after biopsy or present as a delayed complication. Early manifestations include a palpable, pulsatile mass at the biopsy site, whereas delayed presentations may involve recurrent bleeding, ecchymosis, or a persistent cystic mass [40].

In our case, the PSA was incidentally detected on a post-biopsy MRI performed 3 days after the procedure, while the patient reported progressive swelling and discomfort in the affected breast.

### Imaging findings

US represents the imaging modality of choice for the diagnosis and characterization of breast PSAs with an accuracy of more than 95%-100% [1,3,40,41]. On B-mode US, the lesion typically appears as a cystic or mixed-echoic, well-circumscribed mass near the biopsy site, often contiguous with a feeding artery [1,40]. Color doppler imaging demonstrates turbulent flow and often the characteristic “Yin-yang” swirling flow pattern within the aneurysm sac, while color Doppler evaluation at the neck might show the typical “to-and-fro” waveform, corresponding to systolic inflow and diastolic outflow between the PSA and the parent artery [1,16,38].

Ultrasound is usually sufficient for diagnosis, while additional imaging is reserved for selected cases. Mammography may demonstrate a circumscribed dense mass corresponding to the hematoma [40]. Just as in our case, angio-MRI can provide further characterization, with findings varying according to flow dynamics and thrombus organization [1,38]. PSAs may appear in different intensities on native T1- and T2-weighted images depending on the presence of hemorrhagic content, or exhibit flow voids when high-velocity flow predominates [1,16]. After contrast administration, avid enhancement is usually observed within the sac and along the feeding artery, allowing identification of its vascular origin in some cases [1,16].

### Treatment options

Because of the absence of vascular walls, PSAs carry a risk of rupture and hemorrhage, necessitating prompt recognition and management [3,38]. Small (<1 cm), asymptomatic PSAs with a narrow neck may thrombose spontaneously, particularly in patients not receiving anticoagulation, allowing for conservative management with close follow-up [11,12,22,30,34]; manual compression against a firm surface may further promote thrombosis [23].

Definitive treatment depends on size, morphology, location, associated hematoma, symptoms, and hemodynamic stability, with available options summarized in Table 1. Although experience with breast PSAs is limited, these strategies are well established in femoral PSAs, from which most evidence on efficacy and recurrence is derived.

**Table 1:** Treatment options for breast pseudoaneurysms.

	Cases	Indication	Advantage	Disadvantage/ limitations	Efficacy *(a)*	Recurrence rate *(a)*
Observation with/without external compression	22, 11-13, 30, 34	First-line; <1 cm, narrow neck, asymptomatic, no RF	• Non-invasive • No procedural risk • Cost-efficient	• Limited efficiency due to mobile breast tissue • Limited efficacy for large or wide-necked PAs • Careful monitoring • Less effective in AC	Variable depending on clinical setting and morphology	High, but no exact data
US-guided compression	17, 24	First-line; <1 cm, narrow neck, no RF	• More efficient • Non-invasive • No procedural risk • Cost-efficient	• Operator-dependent • Time consuming • Limited efficacy for large or wide-neck PSA	75-98%	30% 41-89% (AC)
US-guided thrombin injection	9, 18, 19, 27, 29, 37, 38	Second-line; > 1 cm, narrow neck	• Mini-invasive • Fast • Effective in AC • Rare complications • Cost-efficient	• Caution if wide neck PSA, AV fistula, multilobulated morphology, infection	94-100%	6%
Percutaneous injection with alcohol	8	Second-line; 1 cm, narrow neck	• Mini-invasive • Fast • Cost-efficient	• Limited evidence • Potential local tissue irritation	No exact data.	No exact data.
Embolization	7, 31, 36	Second-line; > 1 cm or refractory PA, significant hemorrhage	• Effective in complex/high-flow lesions • Precise occlusion	• Technically demanding • Risk of coil migration or distal embolization • Costly	90%	<1%
Surgical repair	6, 10, 14, 15, 16, 20, 21, 23, 25, 26, 28, 32, 35	Last-line; large/refractory PA, expanding hematoma, failed previous treatments	• Definitive treatment • Direct arterial repair • Hematoma evacuation	• Invasive • Infection risk • Requires general anesthesia • Longer recovery • Costly	100%	<1%

*(a):* derived rates from femoral pseudoaneurysms

AC: anticoagulation, AV: Arterio-venous, PSA: Pseudoaneurysm, RF: Risk factor.

*US-guided manual compression* represents a first-line treatment for small PSAs (<1 cm) and post-biopsy hematomas [1]. It achieves thrombosis through stasis in approximately 75%-98% of cases and is typically applied in 20-30 minutes intervals of direct pressure [40,42]. This approach is safe and non-invasive and allows real-time observation of the parent vessel without risk of occlusion. However, it can be time-consuming, uncomfortable for the patient, and less effective in larger or wide neck PSAs [1,43]. Its success rate in the breast is generally lower than in other locations, given the difficulty of maintaining adequate compression on pliable, mobile tissue [44], with reported recurrence rates of around 30% overall and up to 41%-89% in anticoagulated patient [43]. Follow-up color Doppler US is required to confirm complete thrombosis [1]. If unsuccessful, further interventional or surgical treatment may be necessary [6,8,20].

*US-guided percutaneous thrombin* is generally considered a second-line treatment after failed manual compression or in larger PSAs (>1 cm) [44]. It is a minimally invasive, cost-efficient and highly effective option, with reported success rates of up to 94%-100% [[43], [44], [45]]. The procedure is straightforward, typically performed under local anesthesia at the bedside, and provides rapid thrombosis with minimal discomfort, including in anticoagulated patients [1,43]. Complications are rare (<1%) and largely extrapolated from femoral PSA experience, including anaphylaxis to bovine thrombin, systemic coagulopathy, and distal embolization, although none have been reported in breast PSAs [43]. Late reperfusion has been described in up to 6% [43]. Caution is advised in PSAs with a short or wide neck (>1 cm), arteriovenous fistula, multilobulated morphology, or overlying infection, as these features increase the risk of failure or adverse events [43]. In our case, thrombin injection resulted in complete and durable occlusion without recurrence.

*US-guided percutaneous ethanol injection* is a less common alternative for small PSAs, but has proven effective and, in some centers, more cost-efficient [1,8]. Injection of 95% ethanol induces endothelial necrosis, fibrosis, and thrombosis of the feeding artery, but may cause local pain or tissue irritation [1,8]. Similar to thrombin injection, this procedure requires only local anesthesia, usually without hospitalization, and leaves no residual material that could interfere with future breast imaging [8].

*Endovascular or percutaneous embolization* using gelfoam, glue, or coils can be considered for larger or refractory PSAs with extensive hematomas (up to 5 cm) [7,31,36,44]. It is effective in up to 90% of cases, with low recurrence rates (<1%), but is more technically demanding and associated with potential risks such as embolization, coil migration, or rupture [1,46].

*Open surgical repair* is now reserved for selected cases such as large PSAs with wide necks, rapidly expanding hematomas, failed non- or minimal invasive treatments, or when surgery is indicated for another indication [1,43,44]. It involves hematoma evacuation and arterial repair under general anesthesia [43]. Although highly effective with success rates approaching 100% and recurrence rates <1%, it is associated with higher morbidity, including infection, bleeding, and longer hospitalization [1,43,47].

### Prevention

To minimize vascular complications, pre-procedural color Doppler ultrasound of the lesion and surrounding tissue may help identify and avoid prominent vessels [33,35]. When clinically feasible, temporary discontinuation of anticoagulant or antiplatelet therapy prior to breast biopsy should also be considered [43].

## Conclusion

Core needle biopsies and VABB are widely used and recognized as safe, minimally invasive diagnostic procedures with a very low incidence of significant adverse events. Although PSA formation is exceptionally rare, it should be considered in patients presenting with new-onset pain, swelling, or a pulsatile mass after biopsy. Prompt US evaluation allows for accurate diagnosis and characterization. Management should be individualized based on clinical and imaging findings, ranging from conservative observation to percutaneous intervention or, in selected cases, surgical repair.

## Authors' contributions

Antonia Berz: Reporting radiologist, primary author of the manuscript, preparation of figures and tables. Jana Di Vincenzo: Reporting radiologist and supervisor, critical revision and editing of the manuscript. Georgia Tsoumakidou: Interventional radiological procedure, critical revision and editing of the manuscript. Guillaume Fahrni: Reporting radiologist and supervisor, critical revision and editing of the manuscript. Rami Hajri: Reporting radiologist and supervisor, critical revision and editing of the manuscript. All authors approved the publication of the manuscript.

## Patient consent

Written informed consent was obtained from the patient for the publication of case details and images.
